# Towards efficient osteoporosis screening: a simple and practical approach using OSTA and FRAX risk factors

**DOI:** 10.1007/s00774-026-01729-9

**Published:** 2026-05-03

**Authors:** Chiaki Horii, Sakae Tanaka, Toshiko Iidaka, Saeko Fujiwara, Masayuki Iki, Yasushi Oshima, Shigeyuki Muraki, Hiroyuki Oka, Hiroshi Kawaguchi, Kozo Nakamura, Toru Akune, Noriko Yoshimura

**Affiliations:** 1 Department of Orthopaedic Surgery, Faculty of Medicine, The University of Tokyo Hospital, 7-3-1, Hongo, Bunkyo-ku, Tokyo, Japan; 2 Department of Orthopaedic Surgery, Saitama Red Cross Hospital, Saitama, Japan; 3 Department of Preventive Medicine for Locomotive Organ Disorders, 22nd Century Medical and Research Center, the University of Tokyo Hospital, Tokyo, Japan; 4 Department of Pharmacy, Yasuda Women’s University, Hiroshima, Japan; 5 Department of Hygiene and Public Health, Faculty of Medicine, Kansai Medical University, Osaka, Japan; 6 Division of Musculoskeletal AI System Development, Faculty of Medicine, The University of Tokyo, Tokyo, Japan; 7 Department of Orthopaedic Surgery, Nadogaya Hospital, Chiba, Japan; 8 Department of Orthopaedic Surgery, Towa Hospital, Tokyo, Japan; 9 Department of Orthopaedic Surgery, National Rehabilitation Center for Persons with Disabilities, Saitama, Japan

**Keywords:** Osteoporosis, Osteopenia, Screening, OSTA, FRAX

## Abstract

**Introduction:**

Osteoporosis is a major public health concern, particularly in the aging population, where fragility fractures significantly impact quality of life and mortality. Despite the importance of early detection, screening rates for osteoporosis in Japan remain low. The Osteoporosis Self-Assessment Tool for Asians (OSTA) and Fracture Risk Assessment Tool (FRAX) are noninvasive screening tools. This study aimed to develop an efficient method for screening osteopenia using these tools.

**Materials and methods:**

We analyzed data from 1060 Japanese women aged 40–74 years from the Research on Osteoarthritis/Osteoporosis Against Disability (ROAD) study. The OSTA and FRAX scores, as well as individual FRAX risk factors, were assessed for their ability to detect osteopenia. As FRAX score calculations are not publicly available, we evaluated the discriminative ability of the seven risk factors individually. Prior fragility fractures were deemed the most critical, and all 64 possible combinations of the remaining six risk factors were analyzed.

**Results:**

OSTA had the highest discriminative ability (AUC = 0.81), followed by FRAX-Hip (AUC = 0.79) and FRAX-Major (AUC = 0.77). The sensitivity and specificity of OSTA ≤ –1 were 83.7% and 62.6%, respectively. The most effective criterion was OSTA ≤ –1 or at least one FRAX risk factor, achieving a sensitivity of 90.4%, specificity of 47.2%, and positive predictive value of 63.3%.

**Conclusion:**

A simple, noninvasive osteopenia screening method combining OSTA ≤ –1 and FRAX risk factors identified at-risk individuals effectively. This approach could enhance osteoporosis screening in Japan and facilitate early intervention and fracture prevention.

**Supplementary Information:**

The online version contains supplementary material available at 10.1007/s00774-026-01729-9.

## Introduction

Osteoporosis is a common systemic skeletal disorder characterized by decreased bone strength and increased risk of fractures [1] and is a major health issue worldwide [2, 3]. Fragility fractures resulting from osteoporosis not only significantly reduce activities of daily living (ADL) and quality of life (QOL) [4–6] but also worsen life expectancy [7–9]. In Japan, the number of patients with osteoporosis is increasing because of an aging society, and it is estimated that nearly 16 million people are affected [10]. Given the rapidly aging population of Japan, the prevention of osteoporosis and fragility fractures is an urgent public health concern.

Early detection and treatment of osteoporosis before the occurrence of fractures could help prevent osteoporosis-related fractures and the associated decline in ADL. However, osteoporosis and its precursor, osteopenia, are often asymptomatic, leading to a low rate of medical consultation [11]. Therefore, community-based screening programs are essential for the early identification of at-risk individuals. However, the nationwide implementation rate of osteoporosis screening in Japan remains extremely low, averaging only 5.0% [12]. Some guidelines worldwide recommend dual-energy X-ray absorptiometry (DXA) scanning for all women aged ≥ 65 years [13–15]; however, its implementation in Japan remains challenging owing to the availability of DXA and cost considerations.

Therefore, establishing an evidence-based, practical screening method for osteoporosis is of utmost importance in addressing this gap. The Osteoporosis Self-Assessment Tool for Asians (OSTA) [16] and the Fracture Risk Assessment Tool (FRAX) [17] are well-established noninvasive tools for assessing the risk of osteoporosis and fragility fractures, respectively, making them suitable for use in screening programs. This study aimed to develop an efficient screening method to identify individuals with osteopenia using these tools, thereby facilitating timely treatment and prevention strategies. Data from the Research on Osteoarthritis/Osteoporosis Against Disability (ROAD) study baseline survey performed in 2005–2007 were used in this study.

## Materials and methods

### Participants

The ROAD study, initiated in 2005, is a large-scale, population-based cohort study designed to establish epidemiological indicators of bone and joint disorders. It included three cohorts representing distinct geographical areas in Japan: a mountainous region in Hidakagawa, Wakayama, a coastal region in Taiji, Wakayama, and an urban area in Itabashi, Tokyo. Further details of the study design have been documented in prior reports [10, 18]. For this study, data were drawn from a baseline survey of the ROAD study conducted between 2005 and 2007, specifically focusing on participants from the mountainous and coastal regions. Residents aged 40 years and older from these areas were invited to participate in the study through public notices disseminated by local government authorities. The eligibility criteria required individuals to (1) be able to walk to the survey clinic, (2) independently provide self-reported information, and (3) understand and sign an informed consent form. No predefined exclusion criteria were applied to participate in the study.

All participants provided written informed consent prior to their enrollment. The study was approved by the ethics committees of the University of Tokyo (approval nos. 1264 and 1326), the Tokyo Metropolitan Institute of Gerontology (approval no. 5), and Wakayama Medical University (No. 373). Measures were taken to ensure the participants’ safety throughout the study period.

### Questionnaire, interview, and anthropometric measurements

Participants completed a detailed questionnaire administered by an interviewer to evaluate lifestyle factors, such as occupation, personal medical history, family health history, and all questions required to calculate the FRAX [17]. Anthropometric measurements included height, body weight, and body mass index (BMI, kg/m^2^).

### Bone mineral density (BMD) measurement

BMD was assessed in the lumbar spine (L2–L4) and proximal femur using DXA (Hologic Discovery; Hologic, Waltham, MA, USA). The same equipment was used throughout the study, with daily scans of the spine phantom to monitor machine performance across the study regions to ensure consistency in the measurement quality. The phantom’s BMD was calibrated at 1.032 ± 0.016 g/cm^2^ (± 1.5%) during all assessments. Furthermore, a single well-trained physician (N.Y.) performed all examinations to minimize observer variability.

### Definitions of osteopenia and osteoporosis

Osteopenia and osteoporosis were defined as BMD values of less than 80% and 70%, respectively, of the peak bone mass (YAM, young adult mean) of the same sex, according to the criteria of the Japanese Society for Bone and Mineral Research [19]. Thus, the cutoff values for osteopenia and osteoporosis at the femoral neck were 0.630 g/cm^2^ and 0.551 g/cm^2^, respectively, which corresponded to T-scores of –1.70 and –2.55, respectively. Those at the lumbar spine were 0.809 g/cm^2^ for osteopenia and 0.708 g/cm^2^ for osteoporosis, which corresponded to T-scores of –1.44 and –2.17, respectively [20]. Each participant was determined to have osteopenia/osteoporosis if either the BMD at the femoral neck or lumbar spine was less than the cutoff value.

### OSTA score

The OSTA score was calculated based on the weight in kilograms and age in years using the following expression [16]:

$${\mathrm{OSTA}} = {{\left[ {{\mathrm{body}}\,{\mathrm{weight}}\left( {{\mathrm{kg}}} \right) - {\mathrm{age}}\left( {{\mathrm{years}}} \right)} \right]} \mathord{\left/ {\vphantom {{\left[ {{\mathrm{body}}\,{\mathrm{weight}}\left( {{\mathrm{kg}}} \right) - {\mathrm{age}}\left( {{\mathrm{years}}} \right)} \right]} {5\,\left( {{\mathrm{truncated}}\,{\mathrm{to}}\,{\mathrm{yield}}\,{\mathrm{an}}\,{\mathrm{integer}}} \right)}}} \right. \kern-0pt} {5\,\left( {{\mathrm{truncated}}\,{\mathrm{to}}\,{\mathrm{yield}}\,{\mathrm{an}}\,{\mathrm{integer}}} \right)}}$$

In this study, for simplicity, we defined the OSTA index as follows, which is equivalent to OSTA.

$${\mathrm{OSTA}} - {\mathrm{index}} = {\mathrm{age}}\left( {{\mathrm{years}}} \right) - {\mathrm{body}}\,{\mathrm{weight}}\left( {{\mathrm{kg}}} \right)$$

OSTA has been reported to indicate a moderate risk of osteoporosis when it is ≤ –1 [16]. When expressed as the OSTA index, a value of ≥ 5 corresponded to a moderate risk of osteoporosis.

### FRAX score

Height, body weight, and the following dichotomized risk factors were required to calculate the FRAX scores [17]:

Prior fragility fractures.

Parental history of hip fracture.

Current tobacco smoking status.

Long-term use of oral glucocorticoids.

Rheumatoid arthritis.

Other causes of secondary osteoporosis (e.g., type 1 diabetes, osteogenesis imperfecta, untreated long-standing hyperthyroidism, hypogonadism or premature menopause, chronic malnutrition or malabsorption, and chronic liver disease).

Daily alcohol consumption of three or more units per day.

We calculated the FRAX scores for both major osteoporotic fractures (FRAX-Mj) and hip fractures (FRAX-Hip) without using BMD.

### Sensitivity, specificity, and positive predictive values (PPV)

Sensitivity, specificity, and PPV were calculated based on the following standard definitions:

- Sensitivity = Number of individuals with a positive test result divided by the total number of individuals with the disease.
- Specificity = Number of individuals with negative test results divided by the total number of individuals without the disease.
- PPV = Number of individuals with a positive test result who actually have the disease (true positives) divided by the total number of individuals with a positive test result. The values were expressed as percentages.

### Receiver operating characteristic (ROC) curve analysis

ROC curve analysis was performed to evaluate the discriminative ability of each index (OSTA, FRAX-Mj, and FRAX-Hip). The nearest-neighbor method was used to determine the most appropriate cutoff value.

### Statistical analysis

Descriptive statistics were calculated and presented as means with standard deviations (SDs) or frequencies and percentages unless stated otherwise. Differences in proportions were evaluated using the chi-square test, and the Fisher’s exact test was applied when the expected cell size was < 5 cells. All statistical analyses were conducted using STATA software (version 17.0; STATA, College Station, TX, USA).

## Results

A total of 1063 women participated in this study. Three participants who did not undergo BMD measurements were excluded from the analysis. The detailed characteristics of the remaining participants (N = 1060) are shown in Table 1. We divided the participants into three age groups: 40–64, 65–74, and ≥ 75 years, according to the administrative division in Japan. The number of participants with osteoporosis (BMD ≤ 70% of YAM) and osteopenia/osteoporosis (BMD ≤ 80% of YAM) in each age stratum was 54(11.6%)/163(35.1%) in participants aged between 40 and 64 years, 130(37.6%)/243(70.2%) in participants aged between 65 and 74 years, and 163(65.2%) and 232(92.8%) in participants aged 75 years and older, respectively. Owing to the high prevalence of osteopenia in the group aged  ≥ 75 years, we recommend BMD measurement for all females in this age group instead of developing a screening tool for osteopenia. Therefore, participants aged ≥ 75 years were excluded from subsequent analyses.

**Table 1:** Baseline characteristics of the participants

	Age (yrs)			
	40–64	65–74	75–	Total
Number	464	346	250	1060
Age, yrs [SD]	55.1 [6.8]	69.4 [2.7]	79.5 [3.6]	65.5 [11.1]
Height, cm [SD]	154.1 [5.5]	149.8 [5.9]	144.8 [6.1]	150.5 [6.8]
Weight, kg [SD]	54.3 [8.4]	52.3 [8.6]	47.3 [8.3]	52. [8.9]
BMI, kg/m2 [SD]	22.9 [3.4]	23.3 [3.4]	22.5 [3.6]	22.9 [3.5]
Lumbar spine BMD, g/cm^2^ [SD]	0.940 [0.163]	0.831 [0.157]	0.767 [0.173]	0.864 [0.179]
Femoral neck BMD, g/cm^2^ [SD]	0.693 [0.115]	0.601 [0.098]	0.543 [0.097]	0.628 [0.122]
Osteopenia/osteoporosis, no. (%)				
≥ 80% of YAM	301 (64.9)	103 (29.8)	18 (7.2)	422 (39.8)
< 80% of YAM (osteopenia)	163 (35.1)	243 (70.2%)	232 (92.8%)	638 (60.2%)
< 70% of YAM (osteoporosis)	54 (11.6)	130 (37.6)	163 (65.2)	347 (32.7)

*BMI* body mass index; *BMD* bone mineral density; *YAM* young adult mean

Table 2 shows the details of OSTA and FRAX. OSTA ≤ –4 has been reported to be a high-risk factor for osteoporosis, whereas ≤ –1 has been reported to be a moderate risk factor [16], we calculated the numbers and frequencies of participants with both OSTA scores of ≤ –1 and ≤ –4. These corresponded to OSTA index ≥ 5 and ≥ 20, respectively. In the younger group (aged between 40 and 64 years), 38.4%. of the participants had OSTA ≤ –1, and 1.7% had OSTA ≤ –4. In the older group (aged between 65 and 74 years), 90.5% of the participants had OSTA ≤ –1, while 40.8 had OSTA ≤ –4. FRAX scores were calculated without BMD. Overall, 247 participants (30.5%) answered “yes” to at least one of the FRAX risk factors.

**Table 2:** Distribution of OSTA scores and FRAX risk factors among participants

	40–64 yrs	65–74 yrs	Total
N	464	346	810
OSTA			
OSTA (original score)	–0.1 [2.1]	–3.5 [1.9]	–1.6 [2.6]
OSTA-index	0.8 [10.6]	17.2 [9.2]	7.8 [12.9]
OSTA ≤ -1 (OSTA-index ≥ 5)	178	313	491
	38.4%	90.5%	60.6%
OSTA ≤ -4 (OSTA-index ≥ 20)	8	141	149
	1.7%	40.8%	18.4%
FRAX risk factors			
Prior fragility fracture	68	80	148
	14.7%	23.1%	18.3%
Parental hip fracture	26	12	38
	5.6%	3.5%	4.7%
Current smoking	18	11	29
	3.9%	3.2%	3.6%
Glucocorticoids	2	1	3
	0.4%	0.3%	0.4%
Rheumatoid arthritis	9	6	15
	1.9%	1.7%	1.9%
Secondary osteoporosis	28	16	44
	6.0%	4.6%	5.4%
Alcohol	4	3	7
	0.9%	0.9%	0.9%
Presence of any FRAX risk factor	134	113	247
	28.9%	32.7%	30.5%
FRAX scores (%)			
FRAX -Mj	5.3 [3.4]	13.0 [5.3]	8.6 [5.8]
FRAX -Hip	0.6 [0.6]	3.6 [2.7]	1.9 [2.3]

*OSTA* The Osteoporosis Self-Assessment Tool for Asians; *FRAX-Mj* FRAX score for major osteoporotic fractures; *FRAX-Hip* FRAX score for hip fractures

OSTA-index = Age (yrs.)—body weight (kg)

**Fig. 1:**
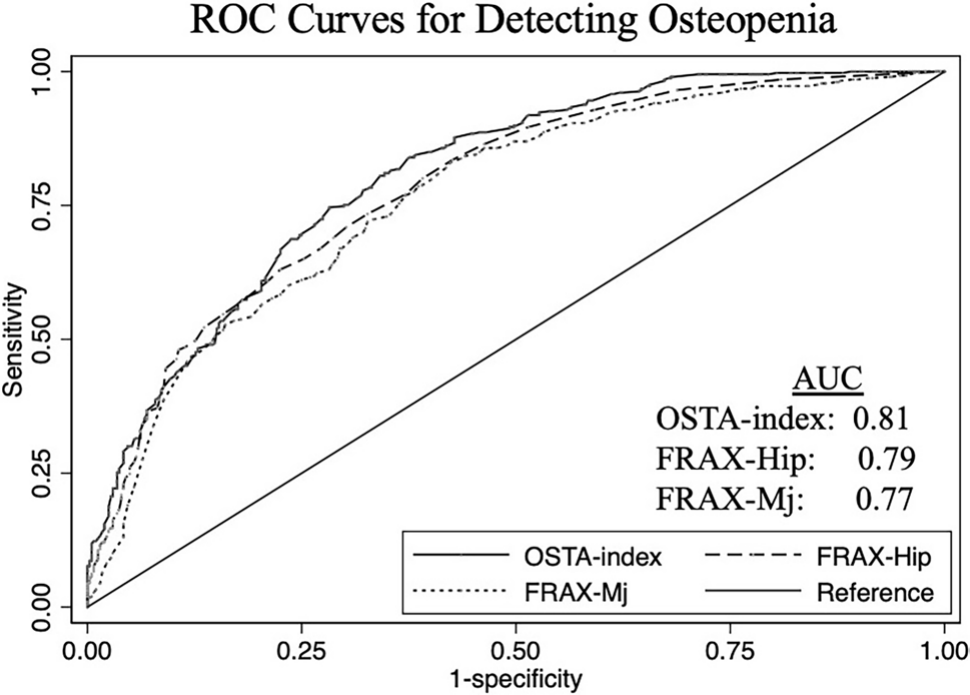
Receiver operating characteristic (ROC) curves for detecting osteopenia ROC curves were used to compare the diagnostic performances of the OSTA index, FRAX-Mj, and FRAX-Hip in identifying osteopenia. OSTA-index = Age (yrs.)—body weight (kg) *FRAX-Mj* FRAX score for major osteoporotic fractures, *FRAX-Hip* FRAX score for hip fractures

Next, we compared the ability of FRAX-Mj, FRAX-Hip, and OSTA index to detect osteopenia using ROC analysis (Fig. 1). The curve (AUC) was 0.81 for the OSTA index, 0.79 for FRAX-Hip, and 0.77 for FRAX-Mj, indicating the highest ability to detect osteopenia of the OSTA index, the second highest of FRAX-Hip, FRAX-Mj, and FRAX-Mj, which remained unchanged, even in the age-specific analysis (Online Resource 1).

Therefore, we focused on the OSTA index. ROC curve analysis of the OSTA index using the nearest method revealed that the most efficient cutoff point for detecting osteopenia was 7.5, which fell between –2 and –1 in OSTA. Here, we annotate that the original OSTA had integer values. We defined the most efficient cutoff value estimated by the nearest method as “the nearest value.” Table 3 shows the details of the AUC, nearest value, sensitivity, specificity of the nearest value, and OSTA ≤ –1 in all age groups and age strata.

**Table 3:** Diagnostic performance of OSTA-index for detecting osteopenia

			OSTA-index ≥ the nearest value		OSTA ≤ –1 (OSTA-index ≥ 5)	
Age	The AUC of OSTA-index	The nearest value of OSTA-index	Sensitivity (%)	Specificity (%)	Sensitivity (%)	Specificity (%)
All	0.81	7.5	74.6	71.8	83.7	62.6
40–64 yrs	0.79	3.0	73.6	70.8	66.3	76.7
65–74 yrs	0.71	17	63.4	68.9	95.5	21.4

*AUC* area under the curve; *OSTA* The Osteoporosis Self-Assessment Tool for Asians

OSTA-index = Age (yrs.)—body weight (kg)

The nearest value represents the most efficient cutoff estimated using the ROC analysis and the nearest method

"OSTA ≤ –1" is a well-established criterion for moderate osteoporosis risk. Our cohort also demonstrated good discriminative performance, with sensitivities and specificities of 84% and 63%, respectively. Therefore, we decided to use "OSTA ≤ –1," or equivalently, "OSTA-index ≥ 5," as the primary criterion (criterion #0) for screening osteopenia. However, this criterion had a relatively low sensitivity of 66% in the younger age group (40–64 years old). To compensate for this, we combined Criterion #0 with the FRAX.

As shown in Fig. 1, both FRAX-Mj and FRAX-Hip exhibited lower discriminative performance than the OSTA index. Furthermore, the algorithm used to calculate FRAX scores is too complicated for use in general population screening and requires internet access, which may limit its applicability in certain settings. Thus, we used the FRAX risk factors without calculating the scores.

FRAX includes seven dichotomized risk factors, of which a history of fragility fracture is the most critical. Therefore, even if the OSTA score is greater than –1, individuals with a prior fragility fracture should still be considered for further examination in osteopenia screening. We defined "criterion #1" as meeting either "criterion #0" or having a prior fragility fracture.

Regarding the remaining six FRAX risk factors, there were 2^6^ (i.e., 64) possible combinations based on whether each factor was included. We calculated the sensitivity and specificity of all 64 combinations for detecting osteopenia (Online Resource 2). Furthermore, similar to the nearest method in the ROC analysis, we examined the combination closest to the ideal point of sensitivity of 1 and specificity of 1. The combination that utilized all FRAX risk factors (criterion #64) was the most efficient. For clarity, the definitions of criteria #0, #1, and #64 are as follows:

- **Criterion #0:** OSTA ≤  − 1 (equivalently, OSTA-index ≥ 5 in our definition)
- **Criterion #1:** Criterion #0 **OR** prior fragility fracture
- **Criterion #64:** Criterion #1 **OR** the presence of **any** of the remaining FRAX risk factors (parental hip fracture, current smoking, glucocorticoid use, rheumatoid arthritis, secondary osteoporosis, and alcohol consumption)

The sensitivities, specificities, and PPV of Criteria #0, #1, and #64 are listed in Table 4. Criterion #64 showed high sensitivity (90.4%) and moderately high PPV (63.3%). In younger participants aged 40–64 years, the sensitivity and specificity were 79.1% and 59.1%, respectively, indicating good sensitivity.

**Table 4:** Sensitivity, specificity, and positive predictive value of screening criteria for osteopenia

Criterion		Sensitivity (%)	Specificity (%)	Positive predictive value (PPV) (%)
#0	OSTA ≤ –1 (OSTA-index ≥ 5)	83.7	62.6	69.2
#1	#0 or prior fragility fracture	87.7	55.9	66.7
#64	#1 or any FRAX risk factors	90.4	47.2	63.3

OSTA, Osteoporosis Self-Assessment Tool for Asia

OSTA-index = Age (yrs.)—body weight (kg)

**Fig. 2:**
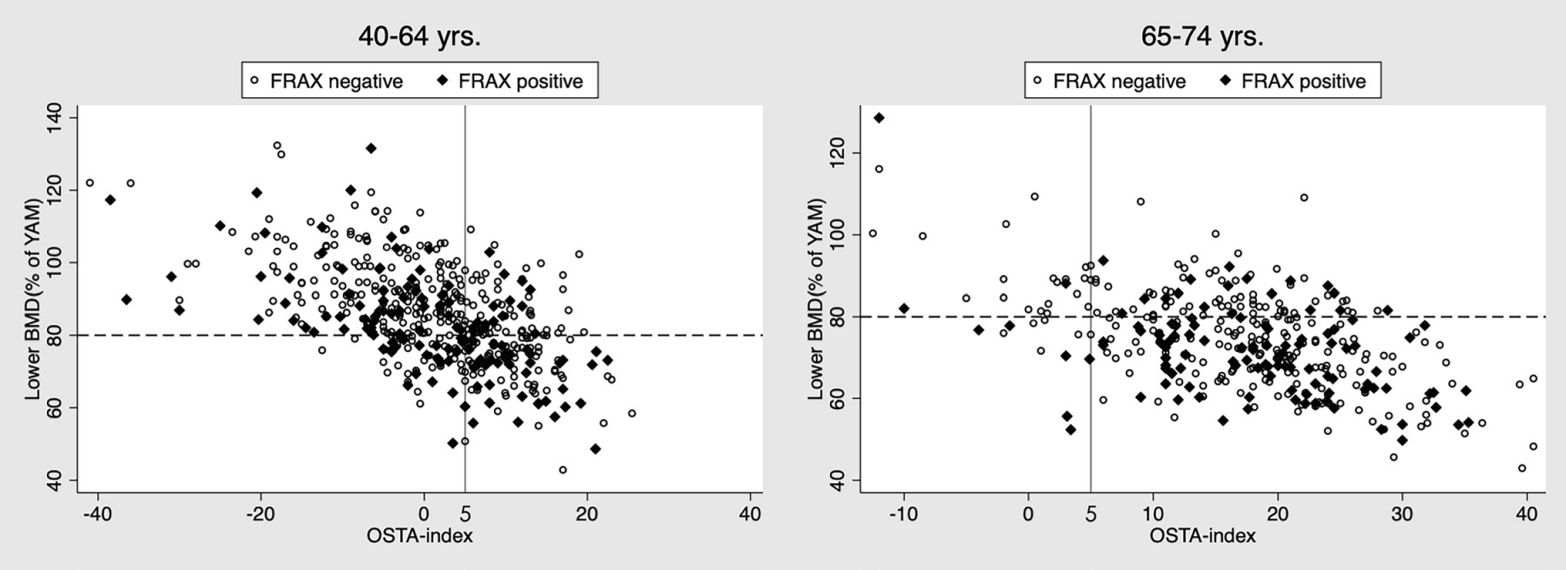
Scatter plots of BMD [12] (% of YAM) and OSTA-index by age group Scatter plots of the BMD (% of YAM) and OSTA index for the 40–64 years (left) and 65–74 years (right) groups. BMD was defined as the lowest % of YAM between the lumbar and femoral neck BMD. White circles represent individuals who were negative for all FRAX risk factors, and black diamonds indicate individuals with at least one positive FRAX risk factor. The dashed horizontal and vertical lines denote the osteopenia and vertical index thresholds, respectively. *BMD* bone mineral density *YAM* young adult mean OSTA-index = Age (yrs.)—body weight (kg)

To validate criterion #64, we created scatter plots of the BMD and OSTA index according to age group (Fig. 2). We used a lower percentage of YAM for lumbar and femoral neck BMD. White circles represent individuals negative for all FRAX risk factors, and black diamonds indicate individuals with at least one positive FRAX risk factor. In this scatter plot, the vertical axis represents the presence or absence of osteopenia, whereas the horizontal axis represents the positive/negative classification according to criterion #0. Specifically:

Bottom-right: True positive.

Top-right: False positive.

Top-left: True negative.

Bottom-left: False negative.

The band-like distribution from the top left to the bottom right reflects the good discriminative ability of the OSTA index for osteopenia. Among the false-negative cases distributed at the bottom left, individuals represented by black diamonds were classified as positive (i.e., true positives) if criterion #64 was applied to them. This indicates an improvement in sensitivity with criterion #64, as shown in Table 4. In the younger age group (40–64 years), 258 participants (55.6%) met criterion #64, whereas in the older age group (65–74 years), 322 (93.1%) fulfilled this criterion; overall, 580 participants (71.6%) screened positive.

## Discussion

This study evaluated the effectiveness of OSTA and FRAX risk factors in screening Japanese women for osteopenia. OSTA index demonstrated the highest discriminative ability (AUC = 0.81), outperforming FRAX-Hip (AUC = 0.79) and FRAX-Mj (AUC = 0.77). Based on these findings, we propose a screening criterion that combines OSTA ≤ –1 with all FRAX risk factors, as using the full set of FRAX risk factors improves sensitivity while maintaining practical applicability.

In the present study, OSTA was superior to FRAX-Mj and FRAX-Hip in detecting osteopenia, which is consistent with previous reports. Chandran et al. and Zhang et al. showed that OSTA outperformed FRAX-Mj and FRAX-Hip in osteoporosis detection in Singapore and China, respectively [21, 22]. Fujiwara et al. reported that an OSTA cutoff of ≤ –1 provided a good balance of sensitivity (88%) and specificity (43%) for detecting BMD at 70% or less of YAM, the criterion of osteoporosis in Japan [19]. The inferiority of FRAX to OSTA in detecting osteoporosis is attributed to its primary aim of assessing fracture risk rather than osteoporosis.

In the present study, we focused on women because OSTA was originally developed for women. However, osteoporosis in men has become an increasingly important public health issue in recent years. We are currently conducting a separate, more detailed evaluation of screening strategies for men and plan to report those results elsewhere. In brief, OSTA also demonstrated better discriminative ability for osteoporosis than FRAX-Major and FRAX-Hip in men. However, particularly in older men, an OSTA cutoff of ≤ –1 achieved high sensitivity at the expense of low specificity. These findings indicate that screening strategies for men may need to consider not only combination with FRAX risk factors but also optimization of the OSTA cutoff.

As shown in Table 3, OSTA showed slightly lower sensitivity in younger age groups (40–64 yrs). However, high sensitivity is the highest priority for minimizing missed cases during screening in the general population. To compensate for this drawback, we used FRAX risk factors. When the presence of one or more FRAX risk factors was included in the criteria, the sensitivity increased as more risk factors were included, whereas the specificity decreased. To balance sensitivity and specificity, we examined the risk factors that should be included using the nearest method (Online Resource2). Our analysis revealed that the criterion utilizing all FRAX risk factors (criterion #64) was the most efficient. Although this criterion has a slightly lower specificity (47.2%), as mentioned above, high sensitivity is crucial for screening. Criterion #64 demonstrated the best sensitivity–specificity balance among all evaluated criteria. Therefore, we considered the use of criterion #64 to be appropriate.

If Criterion #64 is applied in actual screening, it would achieve a sensitivity of 90.4%, enabling the detection of almost all osteopenia cases. By recommending BMD measurements to medical institutions, this approach is expected to contribute to the prevention of osteoporosis and facilitate early intervention, ultimately improving the osteoporosis treatment rate [23]. Because this criterion is intended for primary screening, individuals who screen positive should be referred for confirmatory DXA assessment (lumbar spine and/or femoral neck), clinical evaluation for secondary causes when appropriate, and subsequent management should follow established osteoporosis/osteopenia care pathways. It has been reported that regions with higher implementation rates of osteoporosis screening exhibit lower rates of hemiarthroplasty for hip fractures and lower rates of individuals requiring long-term care [24].

The screening method based on the proposed criterion (Criterion #64) is simple, noninvasive, does not require specialized examinations, and is therefore inexpensive and highly feasible. Following the public dissemination of this criterion, we aimed to assess, on a nationwide scale, whether its adoption would enhance screening rates, leading to higher osteoporosis treatment rates and a reduction in the incidence of fragility fractures.

This study has some limitations. First, the study cohort was not truly representative of the general Japanese population. Specifically, the proportion of smokers was lower than that of the general population [25]. Smoking is also a FRAX risk factor. In this cohort, the sensitivity and specificity for osteopenia detection were 3.4% and 96.3%, respectively. Therefore, when criterion #64 was applied to the general population, the number of positive cases may have increased. However, a significant decrease in specificity is unlikely.

Second, a high proportion of individuals (71.6%) screened positive, while the specificity remained relatively low (47.2%). This is because of the high prevalence of osteopenia (Table 1) and the design of the criteria, which aimed to minimize false negatives. The rationale behind this design is that osteoporosis screening in Japan is currently conducted at 5-year intervals. Considering the expected bone loss over 5 years, it is crucial to detect individuals at the stage of osteopenia rather than osteoporosis and encourage them to seek medical attention early. In the future, a reassessment of these criteria may be warranted if screening is conducted more frequently. Furthermore, compared to the guidelines that recommend DXA screening for all women above a certain age [13–15], the proposed approach may help reduce unnecessary testing.

Third, the data used in this study were obtained in 2005–2007. Changes in lifestyle habits, BMI, and risk factor prevalence over time may affect the applicability of the findings to the current population.

Fourth, the study participants were recruited from rural regions of Japan, which may limit the generalizability of our results to the overall Japanese population, particularly those living in urban settings.

Finally, this was a cross-sectional study design. The long-term effectiveness of screening criteria using the OSTA index and FRAX risk factors, such as their contribution to reducing the actual incidence of fractures, has not yet been evaluated. Further prospective studies are required to validate the clinical utility of these screening criteria. To address this, we plan to assess whether actual fractures can be appropriately predicted by the proposed screening criteria, using longitudinal follow-up data from the ROAD study.

Despite these limitations, the present study showed that simple and effective screening for osteopenia can be performed using OSTA and FRAX. Defining a positive screening result as OSTA ≤ –1 or the presence of any FRAX risk factor yielded a sensitivity of 90.4%, specificity of 47.2%, and PPV of 66.3% for detecting osteopenia.

## Supplementary Information

Below is the link to the electronic supplementary material.

Supplementary file1 (PDF 185 KB)

Supplementary file2 (PDF 44 KB)
