# Supplementary material for: Towards efficient osteoporosis screening: a simple and practical approach using OSTA and FRAX risk factors
Source: J Bone Miner Metab. 2026 May 3;44(4):521–9. doi: 10.1007/s00774-026-01729-9 (PMC13469496; doi:10.1007/s00774-026-01729-9)
Supplement: Supplementary file 1 [file 774_2026_1729_MOESM1_ESM.pdf]

Towards Efficient Osteoporosis Screening: A Simple and Practical Approach Using OSTA and FRAX Risk Factors in *Journal of Bone Mineral and Metabolism*

Supplementary material 1

Receiver Operating Characteristic (ROC) Curves for Detecting Osteopenia in Each Age Stratum

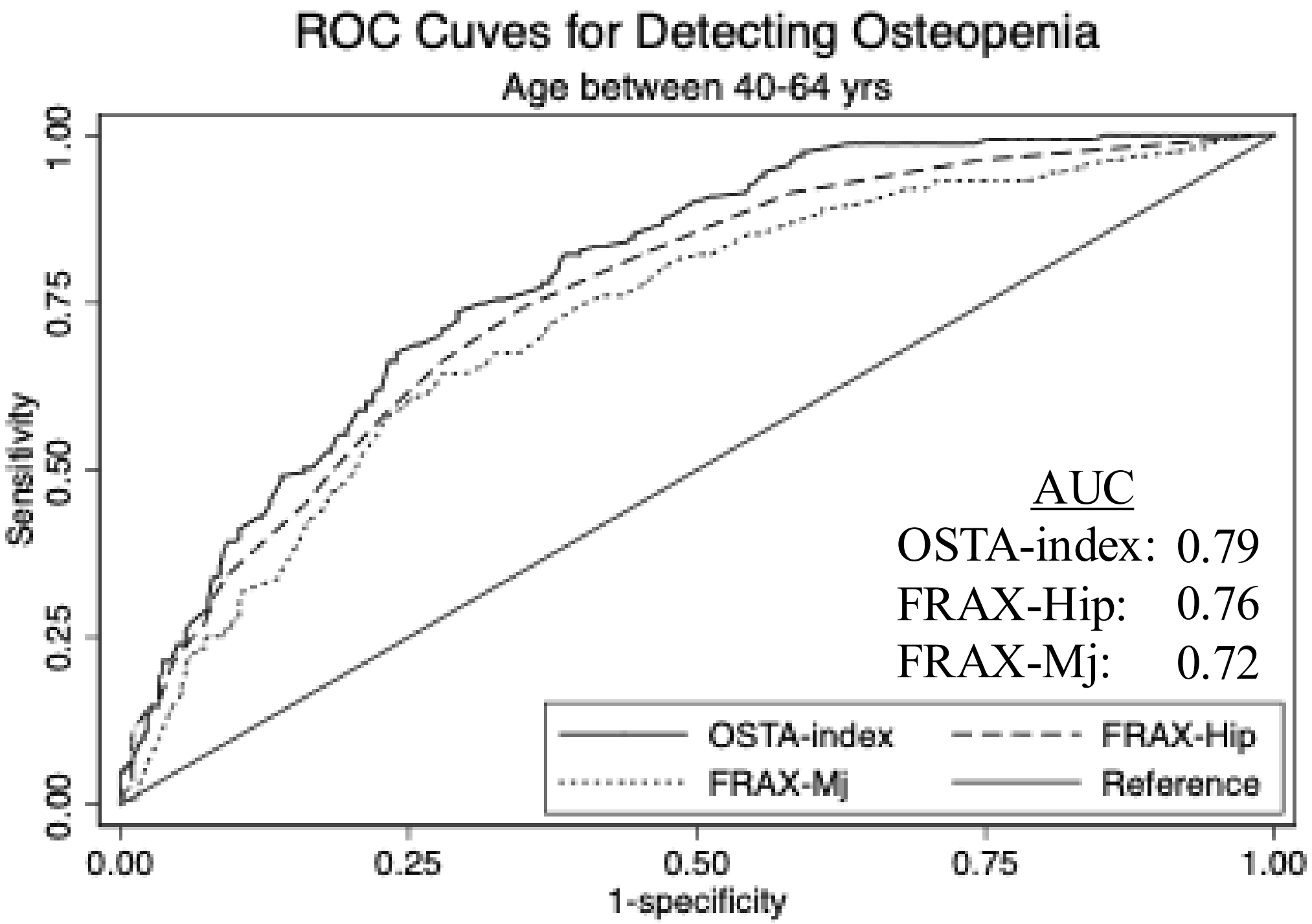

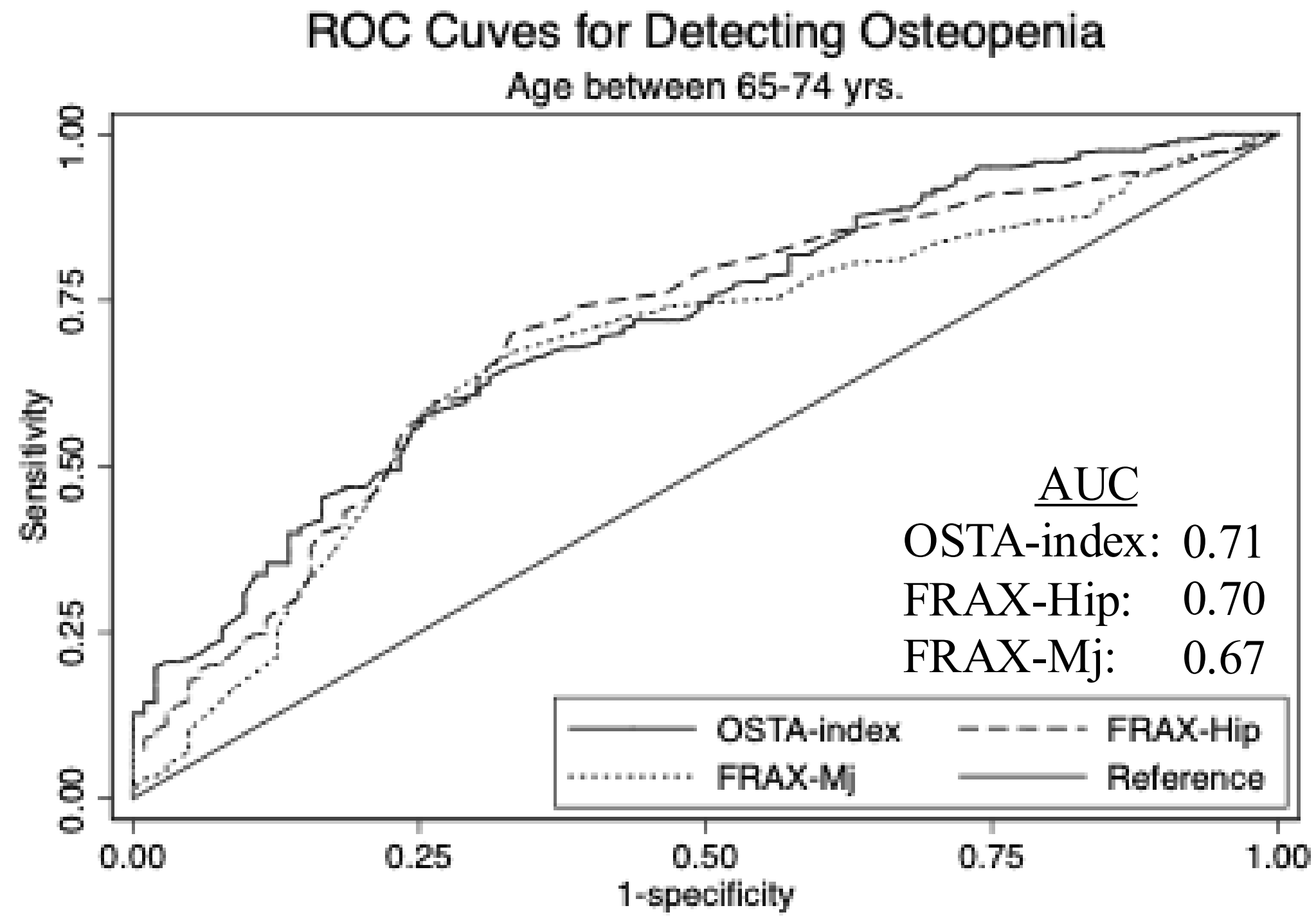

The ROC curves compare the diagnostic performance of the OSTA-index, FRAX-Mj, and FRAX-Hip for identifying osteopenia.
The OSTA-index is defined as follows:

OSTA-index = Age (yrs.) - Body Weight (kg)

Abbreviations:
FRAX-Mj: FRAX score for major osteoporotic fractures
FRAX-Hip: FRAX score for hip fractures

The OSTA-index demonstrated the highest ability to detect osteopenia, followed by FRAX-Hip and FRAX-Mj in both age strata.

Authors: Horii C., Tanaka S., Iidaka T., Fujiwara S., Iki M., Oshima Y., Muraki S., Oka H., Kawaguchi H., Nakamura K., Akune T., and Yoshimura N.

Correspondence: Chiaki Horii, Department of Orthopaedic Surgery, Faculty of Medicine, The University of Tokyo Hospital, Tokyo, Japan.
E-mail: harukabc@gmail.com
