# Supplementary material for: Towards efficient osteoporosis screening: a simple and practical approach using OSTA and FRAX risk factors
Source: J Bone Miner Metab. 2026 May 3;44(4):521–9. doi: 10.1007/s00774-026-01729-9 (PMC13469496; doi:10.1007/s00774-026-01729-9)
Supplement: Supplementary file 2 [file 774_2026_1729_MOESM2_ESM.pdf]

Supplementary material 2

Sensitivity and Specificity for Detecting Osteopenia in Criteria #1 to #64

No.	Sensitivity	Specificity	Distance*	Parental history of hip fracture	Current smoking	Glococorticoidal use	Rheumatoid arthritis	Secondary osteoporosis	Alcohol consumption
1	0.877	0.559	0.573						
2	0.882	0.557	0.569						+
3	0.884	0.53	0.542					+	
4	0.889	0.527	0.539					+	+
5	0.879	0.55	0.563				+		
6	0.884	0.547	0.559				+		+
7	0.887	0.52	0.532				+	+	
8	0.892	0.517	0.529				+	+	+
9	0.879	0.557	0.57			+			
10	0.884	0.554	0.566			+			+
11	0.887	0.527	0.539			+		+	
12	0.892	0.525	0.536			+		+	+
13	0.882	0.55	0.562			+	+		
14	0.887	0.547	0.559			+	+		+
15	0.889	0.52	0.531			+	+	+	
16	0.894	0.517	0.528			+	+	+	+
17	0.882	0.535	0.548		+				

18	0.887	0.532	0.544		+				+
19	0.887	0.505	0.518		+			+	
20	0.892	0.502	0.514		+			+	+
21	0.884	0.525	0.537		+		+		
22	0.889	0.522	0.534		+		+		+
23	0.889	0.495	0.507		+		+	+	
24	0.894	0.493	0.504		+		+	+	+
25	0.884	0.532	0.545		+	+			
26	0.889	0.53	0.541		+	+			+
27	0.889	0.502	0.515		+	+		+	
28	0.894	0.5	0.511		+	+		+	+
29	0.887	0.525	0.537		+	+	+		
30	0.892	0.522	0.533		+	+	+		+
31	0.892	0.495	0.507		+	+	+	+	
32	0.897	0.493	0.503		+	+	+	+	+
33	0.884	0.535	0.547	+					
34	0.889	0.532	0.544	+					+
35	0.892	0.505	0.516	+				+	
36	0.897	0.502	0.513	+				+	+
37	0.887	0.53	0.542	+			+		
38	0.892	0.527	0.538	+			+		+
39	0.894	0.5	0.511	+			+	+	

40	0.899	0.498	0.508	+			+	+	+
41	0.887	0.535	0.547	+		+			
42	0.892	0.532	0.543	+		+			+
43	0.894	0.505	0.516	+		+		+	
44	0.899	0.502	0.513	+		+		+	+
45	0.889	0.53	0.541	+		+	+		
46	0.894	0.527	0.538	+		+	+		+
47	0.897	0.5	0.511	+		+	+	+	
48	0.901	0.498	0.507	+		+	+	+	+
49	0.889	0.51	0.522	+	+				
50	0.894	0.507	0.518	+	+				+
51	0.894	0.48	0.492	+	+			+	
52	0.899	0.478	0.488	+	+			+	+
53	0.892	0.505	0.516	+	+		+		
54	0.897	0.502	0.513	+	+		+		+
55	0.897	0.475	0.486	+	+		+	+	
56	0.901	0.473	0.483	+	+		+	+	+
57	0.892	0.51	0.521	+	+	+			
58	0.897	0.507	0.518	+	+	+			+
59	0.897	0.48	0.491	+	+	+		+	
60	0.901	0.478	0.488	+	+	+		+	+
61	0.894	0.505	0.516	+	+	+	+		

62	0.899	0.502	0.513	+	+	+	+		+
63	0.899	0.475	0.486	+	+	+	+	+	
64	0.904	0.473	0.482	+	+	+	+	+	+

*Distance represents the Euclidean distance to the ideal point where sensitivity = 1 and specificity = 1. It is calculated as the square root of ((1-sensitivity)^2 + (1-specificity)^2).

Sensitivity and specificity for detecting osteopenia were calculated based on whether individuals met the criterion of OSTA _-1 or the presence of a prior fragility fracture, or if they had any of the FRAX risk factors indicated by a "+" in columns E_J of the table.

Article title: Towards Efficient Osteoporosis Screening: A Simple and Practical Approach Using OSTA and FRAX Risk Factors
Journal name: Journal of Bone Mineral and Metabolism
Authors: Horii C., Tanaka S., Iidaka T., Fujiwara S., Iki M., Oshima Y., Muraki S., Oka H., Kawaguchi H., Nakamura K., Akune T., and Yoshimura N.

Correspndence: Chiaki Horii, Department of Orthopaedic Surgery, Faculty of Medicine, The University of Tokyo Hospital, Tokyo, Japan.

E-mail: harukabc@gmail.com
